# Pankun: A New Generation of Broadband Ocean Bottom Seismograph

**DOI:** 10.3390/s23114995

**Published:** 2023-05-23

**Authors:** Dan Liu, Ting Yang, Yizhi Wang, Yuechu Wu, Xinfeng Huang

**Affiliations:** 1 Harbin Institute of Technology, Harbin 150001, China; 12049022@mail.sustech.edu.cn; 2 OBS Laboratory, Department of Ocean Science and Engineering, Southern University of Science and Technology, Shenzhen 518055, China; 3 Shanghai Sheshan National Geophysical Observatory, Shanghai 201602, China

**Keywords:** ocean bottom seismograph (OBS), seismometer, digitizer, leveling system

## Abstract

This paper presents a new broadband ocean bottom seismograph (OBS) developed by the SUSTech OBS lab for passive-source seafloor seismic observations. This instrument, called Pankun, has several key features that set it apart from traditional OBS instruments. In addition to the seismometer-separated scheme, these features include a unique shielding structure to minimize current-induced noise, a compact gimbal for accurate leveling, and low power consumption for extended operation on the seafloor. The design and testing of Pankun’s primary components are thoroughly described in this paper. The instrument has been successfully tested in the South China Sea, demonstrating its ability to record high-quality seismic data. The anti-current shielding structure of Pankun OBS has the potential to improve low-frequency signals, particularly on the horizontal components, in seafloor seismic data.

## 1. Introduction

The ocean bottom seismograph (OBS) is a crucial tool used by seismologists to study earthquakes, the Earth’s interior, and seafloor geodynamic processes [1,2]. However, despite being used for many years, broadband OBSs for long-term passive-source seismology still face various challenges, including frequent recovery failures and poor data quality [3,4,5]. As a result, a major portion of the global seafloor has yet to be surveyed by seismic waves. The lack of high-quality seismic instrumentation in oceanic regions has long been a significant limitation for the field of seismology worldwide [6,7].

**Figure 1:**
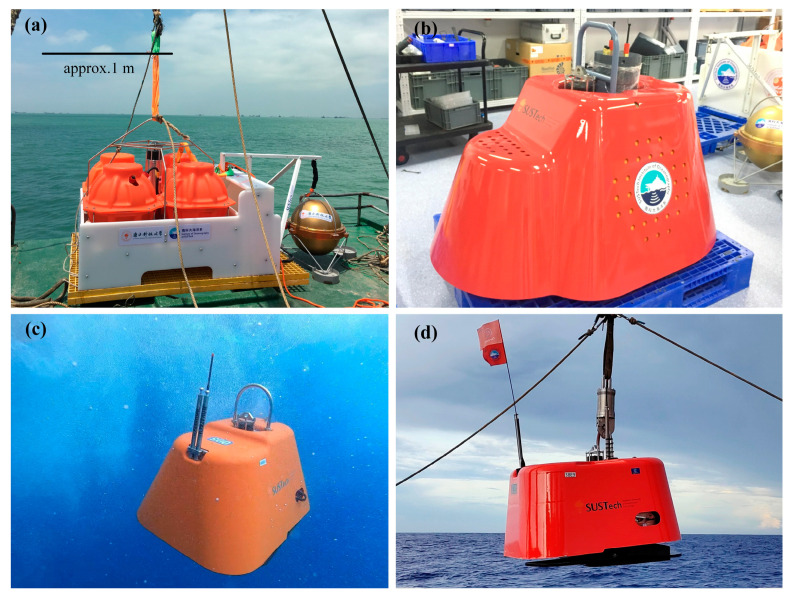
Evolution of passive-source broadband OBS “Pankun” developed by the SUSTech OBS Laboratory since 2016, from (**a**–**d**): the first to fourth generation, respectively.

To address these challenges, the Southern University of Science and Technology (SUSTech) OBS Laboratory has been developing a new generation of passive-source OBSs since 2016. Over the past six years, the team has adopted different strategies and models [8], and the instrument has undergone a four-generation evolution process. It has evolved from traditional multi-sphere, WHOI-style OBSs [9] to a more compact, streamlined, near-cubic shape (Figure 1). The instrument’s name, ‘Pankun,’ is derived from ancient Chinese mythology, where it represents a legendary mighty and enormous fish in the ocean. After several tests, Pankun OBS is close to maturity, with small batch production and deployments for actual seafloor seismic experiments underway.

The performance of Pankun OBS in terms of data quality and ambient noise level has been previously presented in two papers [8,10]. This paper focuses on the technical aspects of the instrument’s development. The article is structured as follows: the Section 2 describes the overall structure and unique features of the instrument, highlighting the designs that suppress current-induced noise; the Section 3 provides a detailed design of the key components, including the sensor module, leveling system, pressure case, data logger, and differential pressure gauge; and finally, we present a brief evaluation of the experimental data recorded by this instrument.

## 2. Pankun OBS’s Structure and Main Features

### 2.1. Overall Structure of Pankun OBS

The primary goal of Pankun OBS is to record earthquake signals on the seafloor for basic seismological research. Like most autonomous OBSs, the Pankun OBS works as follows: the device sinks freely to the ocean bottom and records minuscule seafloor movements due to earthquakes or other sources. A data logger records the digitized data for as long as the battery can power it. Upon recovery, an acoustic signal is sent to the releaser to release the anchor weight, allowing the instrument to ascend to the surface due to its buoyancy. A flashlight and a flag help locate the device. After recovery, the seismic data can be retrieved, processed, and interpreted.

**Figure 2:**
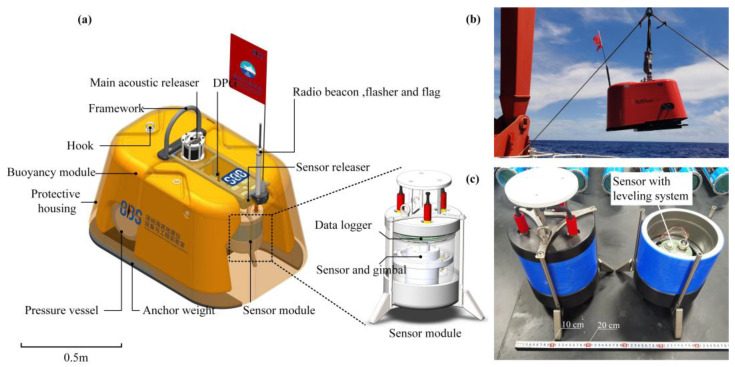
Pankun OBS overall structure. (**a**) A design model of Pankun OBS with the main components labeled. (**b**) Pankun OBS prior to deployment. (**c**) **Left**: Sensor module. **Right**: Interior of a sensor module, featuring a seismometer mounted in a two-axis leveling system.

Figure 2 shows the overall structure of the Pankun OBS. The key elements of Pankun OBS include a framework, a buoyancy module constructed of syntactic foams, a pressure vessel for housing batteries and electronics, a sensor module, the primary acoustic releaser, a sensor releaser, a differential pressure gauge, a radio beacon, a flasher, a protective housing, and an anchor weight.

The framework for the OBS is constructed of 316 L stainless steel and features a semicircular hook on the top. The sensor module, which is suspended from the mainframe using a sensor releaser, includes a digitizer and seismometer as well as a leveling system (as shown in Figure 2b). The buoyancy of the OBS is provided by three blocks of syntactic foam, which are installed on the left and right sides of the framework and above the pressure vessel. These blocks create a hollow space to accommodate the sensor module and provide a buoyancy of approximately 219.8 kg. The sensor is also protected by a protective housing that encloses the entire device and, along with the buoyancy module, forms an almost closed environment for the sensor module. With a safe working water depth of up to 6000 m, the OBS can be deployed on over 99% of the global seafloor.

The descending and ascending speeds of an OBS during deployment and recovery must be carefully designed. A slower descending speed can protect fragile instruments from impact during landing, while a faster ascending speed can save recovery time. These speeds are affected by factors such as the OBS’s weight and buoyancy, as well as its shape, which determines the resistance in the water. Pankun OBS has a total weight of 327.1 kg in air, 38.5 kg in water, and a buoyancy of 25.2 kg without the anchor. This configuration results in descending and ascending speeds of 0.60 m/s and 0.91 m/s, respectively. Testing showed that these speeds are appropriate.

**Figure 3:**
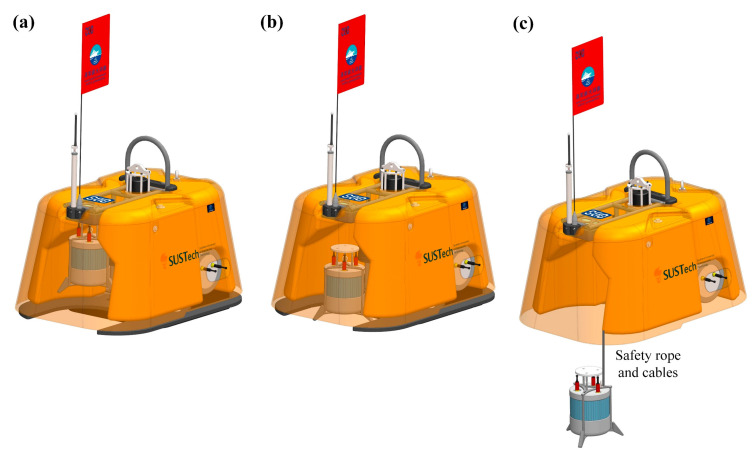
Three modes of operation for Pankun OBS: (**a**) Deployment mode, in which the sensor module is suspended on the sensor releaser and positioned 200 mm above the bottom of the OBS. (**b**) Operating mode, where the sensor releaser is activated upon reaching the seabed to release the sensor module, which then lands on the seabed and connects to the OBS via flexible cables and safety ropes. (**c**) Recovery mode, where the anchor is released by the main releaser and the sensor module is retrieved by the OBS using a safety rope.

Pankun has three operating modes: deployment, operation, and recovery (Figure 3). The center of gravity of the instrument changes as it switches modes. Maintaining balance for proper landing posture during deployment and floating posture on the sea surface during recovery is challenging. To achieve this, the weight and position of each component were carefully adjusted. Our testing shows Pankun maintains a nearly upright posture in each mode. The weight and buoyancy calculations for Pankun can be found in Table 1.

### 2.2. Main Features of Pankun OBS

Pankun OBS has several noteworthy features that can improve its sensor’s coupling with the seafloor and reduce the background noise on its recordings. Unlike the widely used Chinese I-4C OBS [11], Pankun adopts the seismometer-separated scheme, in which the OBS’s sensor module is released from the mainframe after deployment, connecting with the mainframe only through a flexible cable. The separated, relatively smaller sensor module tends to subside into sediments easier, more likely achieving better coupling with the seabed than the integrated OBSs [12]. The module’s small cross-section also reduces the tilt noise generated by currents [13,14,15].

Another feature Pankun has is the shielding structure that completely isolates the sensor module from external bottom currents to minimize noise. Previous sensor shielded OBSs, e.g., the LDEO Trawl Resistant Mount (TRM) and the Scripps Institution of Oceanography Abalone OBS, are only for shallow-water deployments [16], whereas Pankun can operate in the deep ocean up to 6000 m. Our recent laboratory experiment in a circulating water channel shows that shielding can dramatically reduce the noise level on OBS recordings [17]. The actual seismic data collected in the South China Sea also indicates that the noise level has likely been suppressed due to Pankun’s anti-current designs [8,10].

Pankun’s other features include: a low power consumption of 382 mW and an operational duration of over 15 months; a compact leveling system with high accuracy (0.5°) and a large tilt tolerance (34°); a four-channel data logger for a three-component seismometer and differential pressure gauge; a mechanical acoustic releaser for independent instrument recovery; and the use of syntactic foam for flotation, which allows for efficient use of space through customizable shapes.

## 3. Pankun’s Key Components

### 3.1. Sensor Module and the Leveling System

**Figure 4:**
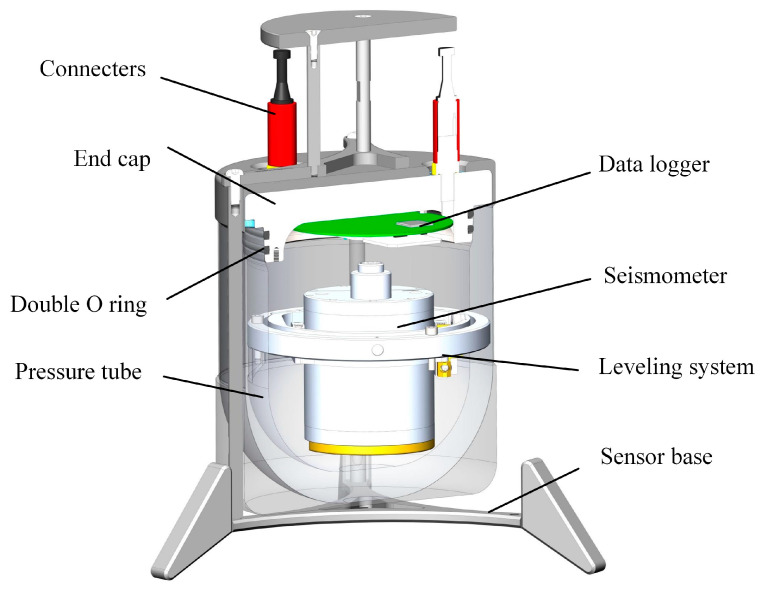
A cut-away view of Pankun’s sensor module with the main components marked.

Pankun’s sensor module, which comprises a seismometer with a leveling system and data logger (Figure 4), is one of the most critical components of the instrument. It is housed in a cylindrical pressure tube with a radius of 0.20 m and a height of 0.25 m, making it very compact and smaller than that of most OBSs. The relatively small size would help it subside into the sediment, achieving a better coupling with the seabed. What is more, a small cross-section area would reduce the influence of the bottom currents and suppress the tilt noise [14].

Pankun’s seismometer is the Trillium Compact from Nanometrics, a widely used seismometer for passive-source OBS due to its small size, low power consumption, and relatively broad frequency range (120 s to 100 Hz) [18].

A seismometer must be meticulously leveled for the optimal recording of three-component seismograms. However, it is typical for a free-fall OBS to incur a tilt angle greater than the necessary 2.5° operating tilt once it reaches the seafloor. Thus, an automatic leveling system is essential for the OBS seismometer to work properly.

**Figure 5:**
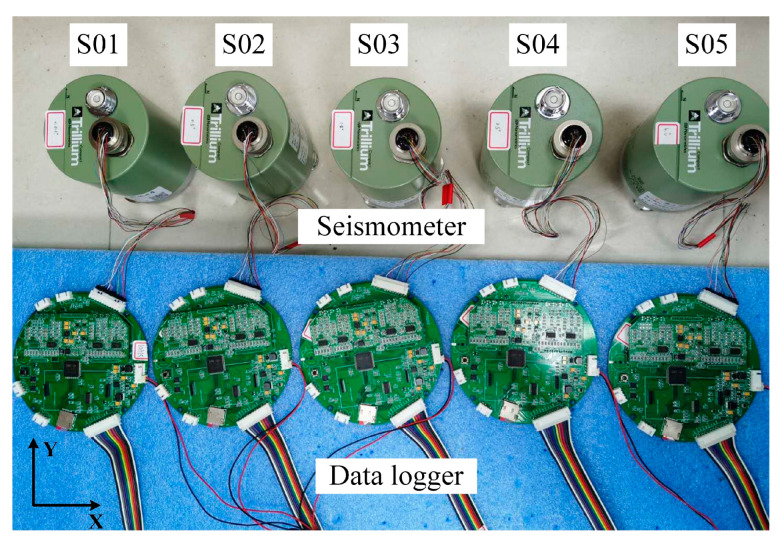
Experiment demonstrating the effect of tilting on seismometer performance. From left to right, the seismometers (S01, S02, S03, S04, and S05) are shown tilted at angles of 0°, 0.5°, 1.5°, 2.5°, and 3.5° along the *X*-axis, respectively. S01 serves as the reference. All seismometers and data loggers used in the experiment are of the same type (Trillium Compact seismometer and Pankun’s digitizer, respectively). The seismometers were adjusted to have identical orientations. The sampling rate for the experiment was 100 Hz, and the input voltage was 7.2 V.

To evaluate the impact of an unleveled seismometer on OBS performance, we conducted an experiment in the laboratory. Five identical seismometers were placed on a platform with gradually increasing tilt angles. We compared the data quality and power consumption of the tilted seismometers to those without tilt using calculations of coherence and power consumption measurements (Figure 5).

**Figure 6:**
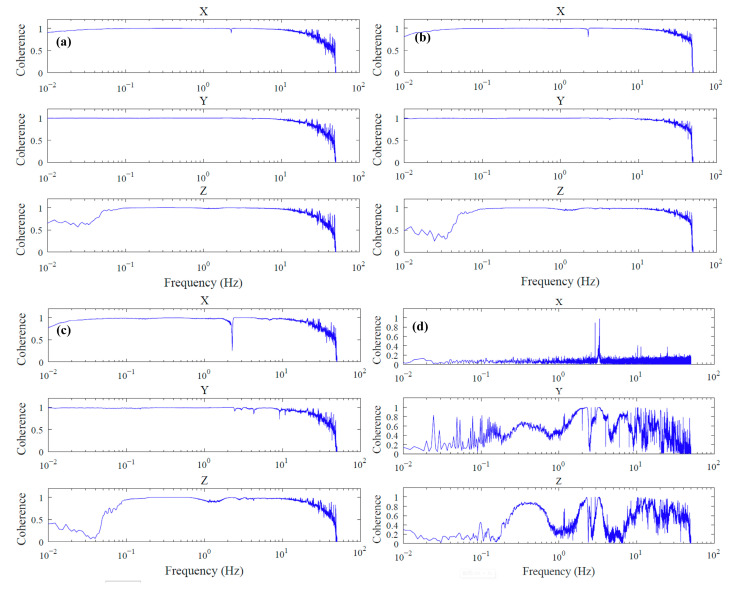
The coherences of three component recordings between S01 and S02 (**a**), S03 (**b**), S04 (**c**), and S05 (**d**), respectively.

Figure 6 illustrates the coherence analysis of the three components between a tilted seismometer and a benchmark seismometer (S01). The analysis was based on one day of seismic data collected at each seismometer and was conducted using Welch’s overlapped averaging periodogram method [19]. The coherence between the recordings of the two seismometers is represented as a function of frequency, and the magnitude-squared coherence is defined as:

(1) $$C_{xy}\left(f\right)=\frac{{\left|P_{xy}(f)\right|}^{2}}{P_{xx}(f)P_{yy}(f)}$$

where $P_{xx}(f)$ and $P_{yy}(f)$ are the power spectral densities of the X and Y components, respectively, and $P_{xy}(f)$ is the cross spectral density of the X and Y components.

The coherence between the first pair (S02 and S01) is the highest for all three components across the observational frequency band (Figure 6a). As the tilt angle increases, the coherence of the other pairs decreases gradually. While the coherences of the Z and X components of S03 and S01 decrease only slightly (Figure 6b), the decrease is more pronounced for S04 and S01 (Figure 6c). The last pair (S05 and S01) shows the worst coherence in the entire frequency band of interest (Figure 6d). This experiment demonstrates that tilting has a significant impact on data quality. If the tilt angle approaches or exceeds 2.5° (the tolerance of the seismometer), the recordings of the seismometer deteriorate. Therefore, the leveling system of OBS should achieve at least 2.5° leveling precision.

**Figure 7:**
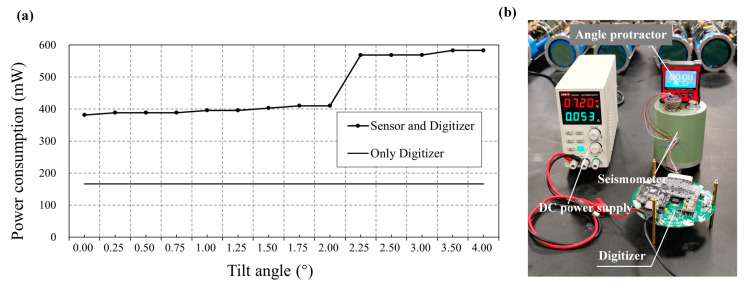
Power consumption for tilted seismometers. (**a**) The power consumption as a function of the seismometer’s tilt angle. (**b**) Photograph of the test setup. The DC power supply provides energy for the system, with an output voltage of 7.2 V. A dual-axis digital angle protector with a 0.01° resolution is used to monitor and adjust the tilt angle of the seismometer. It is necessary to wait until the instrument is stationary before measuring to ensure accurate results.

We also measured the instrument’s power consumption for these tilted seismometers. The power consumption for S01 is 382 mW. It rises to 410 mW for S02 as the tilt angle is around 2°. However, as the tilt angle increases to 2.5°, the system’s power consumption jumps to 554 mW, 45% higher than the leveled S01 (Figure 7). Such an increase in power consumption by a tilted seismometer would significantly reduce the operating duration of the OBS.

It is worth noting that, even though the tilts in this experiment were consistently applied along the X direction, the effects of tilting on data quality and power consumption remain consistent regardless of the tilt direction. This consistency is due to the internal configuration of the Trillium Compact, which utilizes the Galperin configuration [20]. In this configuration, all three sensors are identical and positioned in orthogonal directions (U, V, and W), allowing the conversion to conventional components (X, Y, and Z) [21]. Consequently, the choice of the X direction for the tilt in our experiment is essentially arbitrary within the U, V, and W coordinate systems. Nevertheless, different tilt directions would lead to varying relative effects among the different components.

This experiment shows that the performance of the seismometer is very sensitive to tilting. To record high-quality seismograms and ensure a long operational duration, the seismometer needs to be precisely leveled. The leveling system of Pankun has a small tilt tolerance, and the leveling procedure will be activated if the control system detects the seismometer tilt exceeding 1.5°. This ensures that the seismometer is kept within the optimal tilt range, thus minimizing power consumption and maximizing data quality.

**Figure 8:**
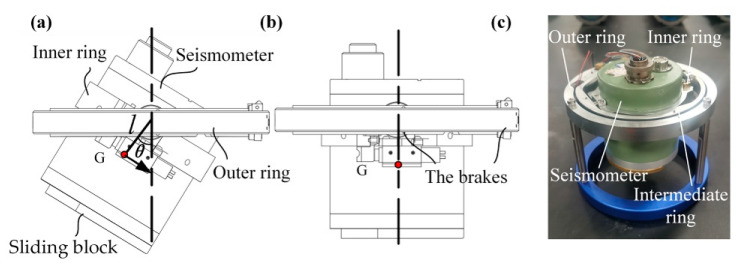
Schematic of the leveling system. (**a**) The seismometer is in a tilt state. The leveling system will reach a new equilibrium state due to gravity when the brakes are unlocked. Note that a sliding block whose position is adjustable is added at the bottom of the seismometer to balance the gravity center of the whole system. (**b**) The leveled and locked seismometer. (**c**) A photograph of the leveling system. The signal wire connected to the data logger from the seismometer uses 14 AWG30 wires to minimize the torque from twisting the wire bundles. The leveling system is mounted inside the pressure tube with the bottom base and supporting rod.

Figure 8 illustrates the leveling system designed for the Pankun OBS. It is a dual-axis device made of aluminum that allows for leveling from a maximum tilt angle of 34° in the vertical direction. The sensor module is very compact: the outer ring is 166 mm in diameter, and its overall height, including the seismometer, is 150 mm. The locking mechanism operates as follows. Before leveling or re-leveling, a brake controlled by a small motor on the gimbal axis is released, enabling the seismometer to undergo passive rotation under the influence of gravity and self-level. Once the leveling process is finished, the brakes are engaged to secure the seismometer in its position. This braking system consists of a steel belt, a wheel, and a motor. When locking, the motor is activated, causing the steel belt to be pulled and firmly hold the brake wheel, ensuring a secure lock of the ring, the seismometer, and the coupling with the pressure tube and sensor base. It is crucial to lock the leveling system before the operation, as any unrestricted movement during transportation and deployment can result in imperfections between the rings, brakes, and pressure tube, leading to nonlinear compliance and negatively impacting data quality. Two Maxon DCX10L brush DC motors drive the brakes, operating through a GPX10A 64:1 gear reducer. These motors are 12 V with a nominal speed of 5990 rpm, a stall torque of 4.36 mN·m, a working current of 0.06 A, and a stall current of 0.2 A.

The leveling system utilizes the self-gravity of the seismometer to achieve leveling. The sensor’s center of gravity is located about its center, 30 mm below the gimbal axes. When the seismometer deviates from the vertical direction, a recovery torque of *mg* × *l* × *sin*(*θ*) will be generated to drive the seismometer to level (where *mg* is the weight of the seismometer, *l* is the distance between the axis and the center of gravity position G, and *θ* is the angle between the line from the center of gravity to the axis and the vertical direction). However, the gravity center of the leveling system does not coincide with that of the seismometer. As a result, the barycenter of this system is not located at the center of the seismometer. It is difficult to accomplish perfect leveling based on the self-gravity of the seismometer alone. Although the tilts of the seismometer in most cases are still within the 2.5° required for the seismometer to work properly, this is not ideal leveling. To tackle this difficulty, we attach an extra sliding block to the seismometer’s bottom. Its position is adjustable. By fine-tuning the extra block, the leveling system will achieve an accuracy of less than 0.5°.

After initial leveling, the seismometer’s dip may increase over time due to its gradual sinking in sediments or the effects of bottom currents. To ensure high-quality seismograms and a long operational duration, a re-leveling scheme is necessary. The control system constantly monitors the seismometer’s status and, if the tilt angle exceeds 1.5°, initiates a re-leveling procedure. The brakes are unlocked, allowing the seismometer to rotate passively under gravity and level itself, and then locked again. This re-leveling process may need to be repeated several times throughout the deployment.

### 3.2. Data Logger

**Figure 9:**
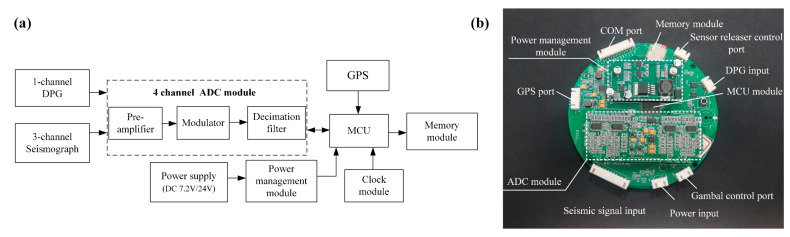
The data logger of Pankun OBS. (**a**) Schematic of the data logger. (**b**) A photograph of the data logger with the main modules marked.

Compared to its counterparts in other passive-source OBS, Pankun’s data logger is characterized by low power consumption (only 166 mW at a 7.2 V input voltage) and high precision. As shown in Figure 9, it consists of four main modules: the Microcontroller Unit (MCU) and storage module, the analog-to-digital converter (ADC) module, the clock module, and the power management module.

The MCU and storage module are the core components of the data logger and are responsible for managing the overall system as well as storing seismic data in memory in miniseed format. The MCU module is a 32 bit STM32 L4 series microcontroller from STMicroelectronics, which is based on a high-performance ARM Cortex-M4 RISC core. It has a micro-USB connector and a working frequency of up to 80 MHz. The data storage medium is a 32 GB micro-SD card, which is interfaced with the MCU through a software driver using a FAT-32 file system.

The ADC module is a 31 bit ADS1281 chip, which is widely used in high-accuracy instrumentation in energy exploration and seismic monitoring applications. It has a dynamic range of 130 dB at a 250 Hz bandwidth and a high accuracy of −122 dB (total harmonic distortion). The converter uses a four order delta-sigma modulator, which provides excellent low-noise levels and linear performance [22]. The digital filter has a data rate that supports outputting 1 to 4000 samples per second, making it sufficient for seismic data acquisition applications. Additionally, the ADS consumes only 12 mW per channel, making it suitable for the low-power requirements of a digitizer. The photograph of the ADC module is shown in Figure 9b.

The clock module is a circuit based on a temperature-compensated crystal oscillator (TCXO), which has a temperature range of −40 °C to +85 °C. It has a frequency stability of ±280 ppb (±0.28 ppm) and generates a stable clock frequency for the ADC and MCU through a phase-locked loop. Overall, the data logger has a low power consumption of only 166 mW at a 7.2 V input voltage and high precision, making it suitable for use in passive-source OBS.

The power management module in Pankun OBS ensures a stable and continuous power supply to all components, including the seismometer, data logger, sensor releaser, and DPGs. Two types of batteries are used in Pankun: a lithium thionyl chloride battery pack (7.2 V) and a rechargeable Li-ion battery pack (24 V). The lithium thionyl chloride battery is chosen for its ability to provide low current and long-term discharge power for the digitizer, seismometer, and DPG. With a capacity of up to 650 Wh/kg at a low release rate, it consists of 60 DD-sized lithium thionyl chloride batteries with a single battery capacity of up to 35 Ah at 3.6 V. On the other hand, the sensor releaser and brake operations require intermittent, high-current power, which is provided by the 18,650 rechargeable lithium-ion (24 V) battery with an energy density of about 300 Wh/kg and consisting of 36 batteries. To generate the voltage required by different modules (12 V for the seismometer, 3.3 V for the MCU, 3.3 V and ±2.5 V for the ADC, 24 V for the sensor releaser, and 7.2 V for the DPG), a DC-DC power management module based on XL6019 is used. This module can work with a DC 5 V to 40 V input voltage with a conversion efficiency of up to 95%. The power management module is integrated into the data logger, as shown in Figure 9b.

### 3.3. Differential Pressure Gauge (DPG)

**Figure 10:**
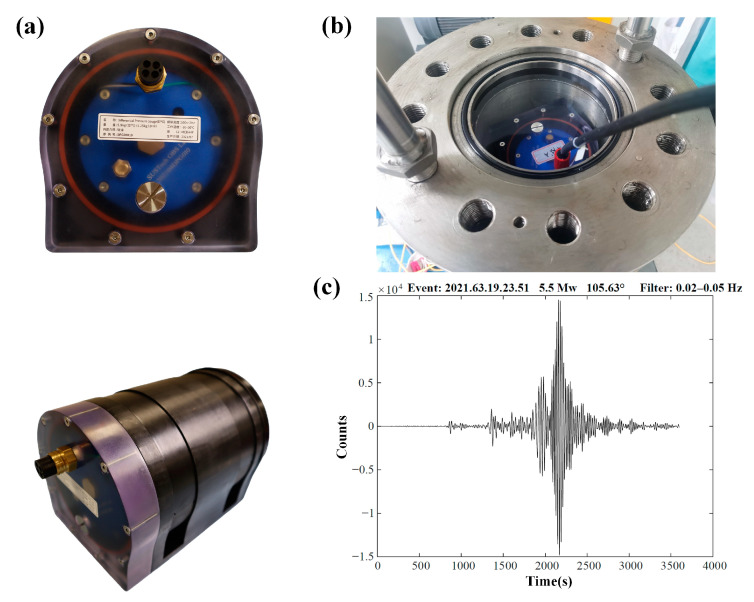
The Differential Pressure Gauge (DPG) shown in (**a**) as a photograph, with a weight of 5.5 kg in air and 1.2 kg in water, measures 247 mm in length and 152 mm in height. (**b**) The DPG is being tested in a pressure tank, withstanding a pressure of 66 Mpa. (**c**) Examples of seismograms recorded by the DPG.

Earthquakes can also cause pressure changes in seawater. Pressure recordings have been an important complement to the seismometer’s recordings in various seismic applications [23]. They have recently been used to remove compliance noise to improve the signal-to-noise ratio for seismic recordings [14]. Therefore, the pressure gauge has been a standard component for modern OBS. The pressure sensor in Pankun OBS is a deep-sea Differential Pressure Gauge (DPG) developed by SUSTech OBS Laboratory based on the theory and method of Cox and Webb [24] (Figure 10).

The DPG, which is filled with silicone oil of viscosity 500 cSt and equipped with a pressure relief valve between the reference and outer chambers, is able to withstand pressures of over 66 MPa (as shown in Figure 10b). The DPG can measure pressure signals across a wide frequency range, from 0.002 Hz to 2 Hz. It features a differential pressure sensor with two chambers located at either end of the sensor. The outer chamber’s pressure changes in response to external pressure and is insulated by a soft rubber film. A capillary leak, measuring 0.3 mm in diameter and 38 mm in length, connects the two chambers. This functions as a high-pass filter [24], with its angular frequency determined by the capillary tube’s diameter and length as well as the silicone oil’s viscosity. However, accurately calibrating the DPG can be challenging due to changes in the silicone oil’s viscosity with pressure and temperature [25]. The typical power consumption of the DPG is 15 mW when using a 7.2 V input voltage, and it can be powered by the same supply as the seismometer.

### 3.4. The Pressure Vessels

The Pankun OBS has two pressure vessels: one for the battery and the other for the sensor module. These vessels must withstand the harsh deep-sea conditions of high pressure and corrosion for the OBS’s long-term operation on the seafloor. Therefore, the choice of material is crucial. Duplex stainless steel, 316 stainless steel, aluminum, and titanium alloy are common materials used for making pressure vessels. The strength-to-weight ratio of stainless steel is low, which means more buoyancy materials are needed for the same strength of the structure. Aluminum alloy 7075 is widely used as the pressure vessel material. The strength-weight ratio is close to that of titanium alloy, which is about twice that of stainless steel. It is a lightweight alloy but sensitive to seawater corrosion. Because the connectors need to be installed on the end cap, if it is made of aluminum alloy, the fixed connector easily damages the anodic oxide layer, resulting in the loss of protection of the aluminum alloy structure. Taking all factors into consideration, we chose TC4 (Ti-6Al-4V) as the pressure vessel material, with a strength limit of 952 Mpa and a yield limit of 879 Mpa. The pressure vessels are designed with a safety factor of 1.8 and sealed with double sealing rings. 

**Figure 11:**
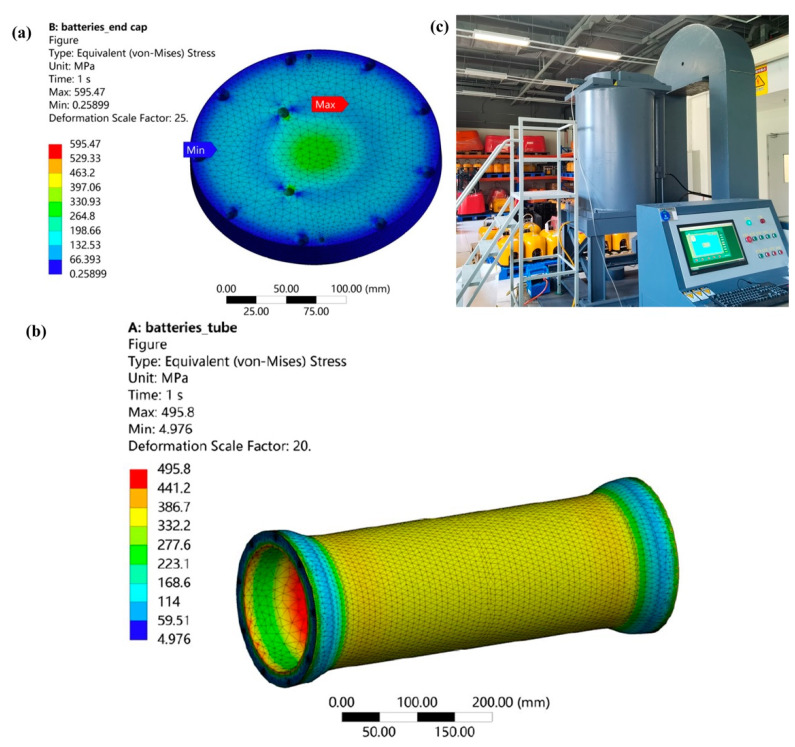
Strength analysis of Pankun OBS pressure vessels at 6000 m depth. (**a**) Finite element analysis of the end cap of the pressure vessel. (**b**) Finite element analysis of the pressure tube of the vessel. (**c**) Actual pressure test of the finished product in a pressure tank at the SUSTech OBS Laboratory.

We simulated the pressure of the deep ocean up to 6600 m and analyzed the strength of both vessels and the end caps using ANSYS software. Figure 11a shows the finite element analysis results of the pressure vessel for the battery under a pressure of 66 Mpa. The two vessels have the same cylinder diameter and thickness. Based on these analyses, we determined the actual parameters of the two vessels, as shown in Table 2.

To ensure that the vessels can properly function at the designed water depths, we conducted a pressure test on the completed products within a pressure tank located in our SUSTech OBS Laboratory (Figure 11c). During this test, a static pressure of 66 MPa was applied to the vessels for a duration of 4 h. The vessels successfully withstood this rigorous test without exhibiting any signs of damage.

## 4. Testing Pankun and Its Data Quality Assessment

**Figure 12:**
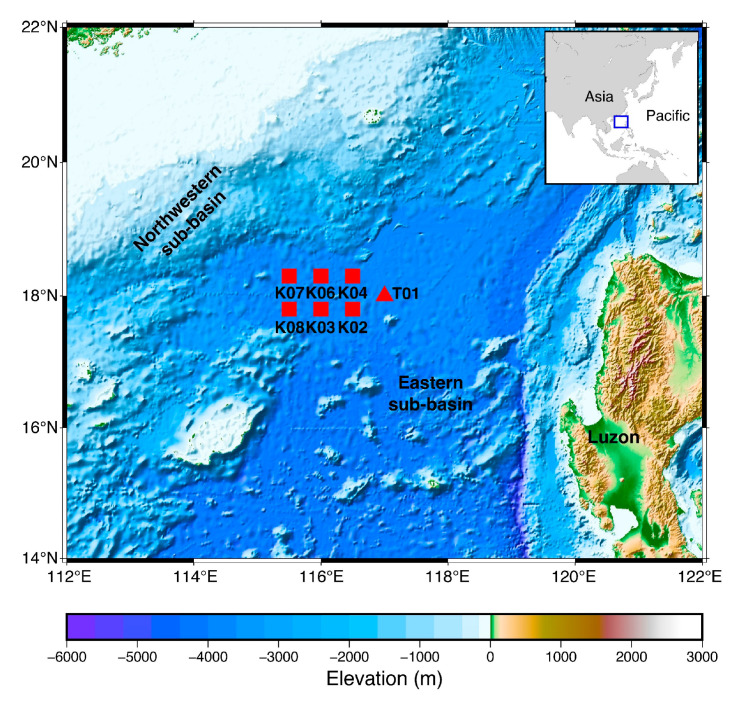
Pankun testing sites in the South China Sea. The deployment durations of OBSs T01, K07, and K06 were short-term, about two weeks. The rest of the OBSs had seven months of deployment.

Seven Pankun OBSs were deployed on the South China Sea (SCS) seafloor in two separate tests in 2019–2020. Figure 12 shows the locations of the test sites. The detailed data quality analyses and the instrument performance assessments, including its noise level, power consumption, seismometer tilt angle, and clock drifting, are presented in Liu et al. (2022) and Wang et al. (2022) [8,10]. Here we simply present a comparison of waveforms recorded by Pankun OBSs and land stations.

In April 2019, we deployed a Pankun in the SCS for the first deepwater test to verify the basic functions of the entire system, including the release system, sinking, and floating speeds, level system, and data recorder. The instrument was deployed at the beginning of the cruise and recovered on the same cruise ship two weeks later. After solving several minor problems revealed in this test, such as the sea surface posture, ascent speed, activation of the re-leveling scheme, etc., in October 2019, we deployed six Pankun OBSs in the northwestern sub-basin of the SCS (Figure 12). Two of them were recovered on the same cruise after two weeks of deployment. In May 2020, seven months later, the rest of the four OBSs were recovered smoothly. Unfortunately, one of the recovered Pankuns, K04, did not return valid data due to a technical assembly defect. The water depth of the above seven instruments is about 4000 m.

**Figure 13:**
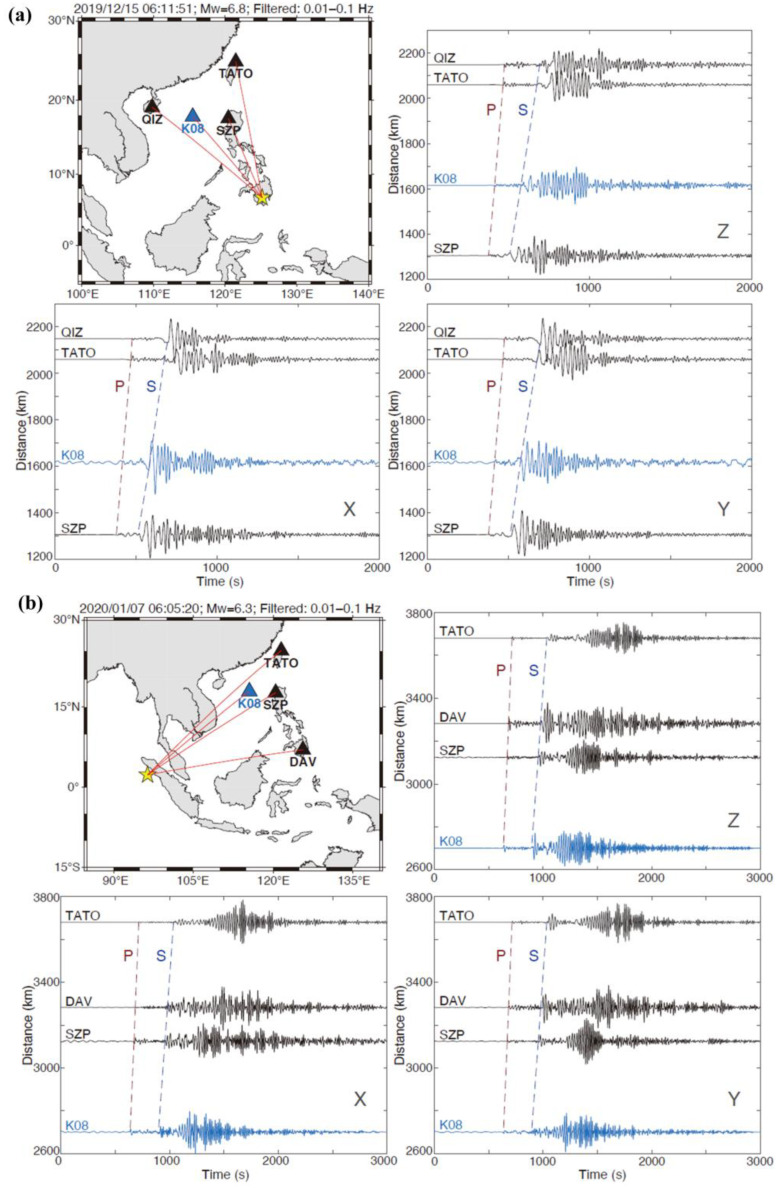
Comparison of three-component seismograms recorded by one of Pankun’s OBSs (K08, blue triangle) and nearby land stations (black triangles) from two earthquakes. The predicted P and S arrivals are marked by red and blue dashed lines, respectively. The seismic waveforms are filtered by a broadband filter (10–100 s). X and Y represent the two horizontal components, and Z is the vertical component. The top left panels show the locations of Pankun’s OBS, land stations, and earthquakes (yellow star). The earthquake information is given on top of the maps. (**a**) Mw6.8 earthquake occurred near the Mindanao, Philippines on 15 December 2019; (**b**) Mw6.3 earthquake occurred near the northern Sumatra, Indonesia on 7 January 2020.

To demonstrate the quality of ocean bottom seismic data recorded by this instrument, we compared the seismograms recorded by Pankun OBSs with those recorded on land stations. Figure 13 shows such comparisons of three component seismograms from two representative earthquakes. It is evident that seismograms at Pankun have comparable quality to those at nearby land stations. Their vertical components are nearly identical, whereas the horizontal components of Pankun are just slightly inferior. Given the high ambient noise and the influences of bottom currents, seismic data with such quality, especially at the horizontal components, appears to be better than, or at least comparable to, that recorded by other OBSs. The data for sites IU-TATO and IC-QIZ was from GSN [26,27].

## 5. Conclusions

The Pankun OBS is a seismometer-detached, low-power, long-duration instrument used for passive source (earthquake) recordings. It has a unique shielding design to isolate its sensor from the bottom currents. A relatively small sensor module would help achieve better coupling with the seabed. It should be noted that the seismometer of Pankun (Trillium Compact) is very sensitive to tilting. A tilted seismometer will affect the instrument’s data quality and increase power consumption. The built-in leveling system can level the seismometer with a maximum tilt level of 34° and an accuracy of 0.5°. The re-leveling procedure will be activated whenever the system detects that the seismometer tilts more than 1.5°. Pankun OBS is equipped with a four channel data logger whose typical power consumption is only 166 mW. In addition to a 3-C seismometer, Pankun OBS also has a DPG sensor, which records water pressure changes. Testing of Pankun OBS indicates that the quality of seismic data recorded by this instrument is comparable to that of its land stations. In particular, the horizontal component seismogram is fairly good, which may be attributed to the unique shielding structure isolating the sensor from the bottom currents.

## Figures and Tables

**Table 1:** Buoyancy calculations for all components of Pankun OBS.

Name	Main Materials	Weight in Air/kg	Buoyancy /kg	Weight in Water/kg
Framework	Titanium alloy	9.1	2.7	6.4
Main acoustic releaser	Duplex stainless steel	26.0	8.0	18.0
Sensor releaser	Electrical pure iron	6.8	1.8	5.0
Sensor module	Titanium alloy (TC4)	17.6	7.0	10.6
DPG	Engineering plastics	5.5	4.3	1.2
Pressure vessel	Titanium alloy (TC4)	44.3	19.5	24.8
Radio beacon and flasher	Titanium alloy	1.4	0.4	1.0
Flag etc.	Engineering plastics	0.2	0.2	0.0
Protective housing	Acrylonitrile Butadiene Styrene plastic	14.9	14.6	0.4
Cables	-	1.0	0.2	0.8
Screws	316 stainless steel	2.9	0.8	2.1
Buoyancy module	Syntactic foam	124.4	219.8	−95.4
Anchor	Carbon steel	73.0	9.3	63.7
Rising	NA	NA	NA	−25.2
Sinking	NA	NA	NA	38.5
OBS	NA	327.1	NA	NA

**Table 2:** The pressure vessel specifications.

Name	Unit	Pressure Vessel (Sensor Module)	Pressure Vessel (Batteries)
Length of pressure tube	mm	198	557
Diameter of pressure tube	mm	210	210
Weight in air	kg	14.3	30.4
Weight in water	kg	7.4	10.5
Maximum depth before failure	m	11,665	11,407
Safety factor	NA	1.8	1.8
Safe working depth	m	6480	6337

## Data Availability

The Pankun OBS data used in this study can be downloaded from the data center of SUSTech OBS Laboratory at https://obslab.sustech.edu.cn/data-1S.html (accessed on 15 May 2023), and the IU-TATO and IC-QIZ data can be obtained from the Incorporated Research Institutions for Seismology (IRIS) Data Management Center at www.iris.edu (accessed on 15 May 2023).

## References

[1] Collins J.A., Vernon F.L., Orcutt J.A., Stephen R.A., Peal K.R., Wooding F.B., Hildebrand J.A. (2001). Broadband seismology in the oceans: Lessons from the ocean seismic network pilot experiment. Geophys. Res. Lett..

[2] Suyehiro K., Montagner J., Stephen R.A., Araki E., Kanazawa T., Orcutt J., Shinohara M. (2006). Ocean seismic observatories. Oceanography.

[3] Liu C., Hua Q., Pei Y., Yang T., Xia S., Xue M., Le B.M., Huo D., Liu F., Hung T.D. (2014). Passive-source Ocean Bottom Seismograph (OBS) array experiment in South China Sea and data quality analyses. Chin. Sci. Bull..

[4] Le B.M., Yang T., John Chen Y., Yao H. (2018). Correction of OBS clock errors using Scholte waves retrieved from cross-correlating hydrophone recordings. Geophys. J. Int..

[5] Hung T.D., Yang T., Le B.M., Yu Y. (2019). Effects of Failure of the Ocean-Bottom Seismograph Leveling System on Receiver Function Analysis. Seismol. Res. Lett..

[6] Shiobara H., Kanazawa T., Isse T. (2013). New Step for Broadband Seismic Observation on the Seafloor: BBOBS-NX. IEEE J. Ocean. Eng..

[7] Fukao Y., Obayashi M., Nakakuki T. (2009). The Deep Slab Project Group Stagnant slab: A review. Annu. Rev. Earth Planet. Sci..

[8] Liu D., Yang T., Manh L.B., Wu Y.C., Wang Y.Z., Huang X.F., Du H.R., Wang J., Chen Y.S. (2022). Seismometer-detached broadband ocean bottom seismograph (OBS): Development, test, and data quality analysis. Chin. J. Geophys..

[9] WHOI Broadband OBS (BBOBS). https://web.whoi.edu/obslab/instrumentation/broadbandobs/.

[10] Wang Y., Yang T., Wu Y.C., Liu D., Huang X.F., Wang J., Zhong W.X., Shou H.T., Zhou Y., Chen Y.S. (2022). A new broadband ocean bottom seismograph and characteristics of the seismic ambient noise on the South China Sea seafloor based on its recordings. Geophys. J. Int..

[11] Hao T.Y., You Q.Y. (2011). Progress of homemade OBS and its application on ocean bottom structure survey. Chin. J. Geophys..

[12] Sthler S.C., Sigloch K., Hosseini K., Crawford W.C., Barruol G., Schmidt-Aursch M.C., Tsekhmistrenko M., Scholz J.R., Mazzullo A., Deen M. (2016). Performance Report of the RHUM-RUM Ocean Bottom Seismometer Network around La Réunion, western Indian Ocean. Adv. Geosci..

[13] Webb S.C. (1998). Broadband seismology and noise under the ocean. Rev. Geophys..

[14] Bell S.B., Forsyth D.W., Ruan Y. (2015). Removing noise from the vertical component records of ocean-bottom seismometers: Results from year one of the Cascadia Initiative. Bull. Seismol. Soc. Am..

[15] An C., Cai C., Zhou L., Yang T. (2022). Characteristics of low-frequency horizontal noise of ocean-bottom seismic data. Seismol. Res. Lett..

[16] Hilmo R., Wilcock W. (2020). Physical sources of high-frequency seismic noise on Cascadia Initiative ocean bottom seismometers. Geochem. Geophys. Geosyst..

[17] Wu Y.C., Yang T., Liu D., Dai Y., An C. (2023). Current-induced noise in ocean bottom seismic data: Insights from a laboratory water flume experiment. Earth and Space Science.

[18] Ringler A.T., Hutt C.R. (2010). Self-Noise Models of Seismic Instruments. Seismol. Res. Lett..

[19] Stoica P., Moses R.L. (2005). Spectral Analysis of Signals.

[20] Graizer V. (2009). The response to complex ground motions of seismom-eters with Galperin sensor configuration. Bull. Seismol. Soc. Am..

[21] Trillium Compact User Guide. https://www-iuem.univ-brest.fr/pops/attachments/1489/trilliumcompact_userguide.pdf/.

[22] High-Resolution Analog-to-Digital Converter. https://www.ti.com/lit/ds/symlink/ads1281.pdf.

[23] Doran A.K., Rapa M., Laske G., Babcock J., McPeak S. (2019). Calibration of differential pressure gauges through in situ testing. Earth Space Sci..

[24] Cox C., Deaton T., Webb S. (1984). A deep-sea differential pressure gauge. J. Atmos. Ocean. Tech..

[25] Schmidt-Aursch M.C., Crawford W.C., Beer M., Kougioumtzoglou I.A., Patelli E., Au S.K. (2015). Ocean-Bottom Seismometer. Encyclopedia of Earthquake Engineering.

[26] Albuquerque Seismological Laboratory/USGS (2014). Global Seismograph Network (GSN-IRIS/USGS).

[27] Albuquerque Seismological Laboratory (ASL)/USGS (1992). New China Digital Seismograph Network.

